# The impact of influenza on the ability to work, volunteer and provide care: results from an online survey of Canadian adults 50 years and older

**DOI:** 10.1186/s12889-022-14581-z

**Published:** 2022-11-18

**Authors:** Nancy M. Waite, Jennifer A. Pereira, Sherilyn K. D. Houle, Vladimir Gilca, Melissa K. Andrew

**Affiliations:** 1 School of Pharmacy, University of Waterloo, 10A Victoria St. S. Kitchener, Ontario, N2G 1C5 Canada; 2 JRL Research & Consulting Inc., 3465 Marmac Crescent, Mississauga, ON L5L 5A1 Canada; 3 Institut national de santé publique du Québec, Laval University, 2400 d’Estimauville, Quebec City, Quebec G1E 7G9 Canada; 4 Department of Medicine (Geriatrics), Dalhousie University, Veterans’ Memorial Building, 5955 Veterans’ Memorial Lane, Halifax, NS B3H 2E1 Canada

**Keywords:** Influenza, Older adults, Work, Volunteer, Caregiver

## Abstract

**Background:**

Influenza is associated with a decline in functional abilities among Canadian older adults, although specific impacts on daily life have not been fully explored.

**Methods:**

In August 2019 and May 2020, we conducted surveys of Canadian adults 50-64 years and 65 years and older through an online market research platform. The survey included questions about the impact of diagnosed influenza or self-reported influenza-like-illness (ILI) on working, volunteering and caregiving.

**Results:**

We surveyed 1006 adults in the 50-64 year age group about the 2018/19 season and 1001 about the 2019/20 season. In the 65 years and older age group, we surveyed 3548 and 3500 individuals about the 2018/19 and 2019/20 influenza seasons, respectively. In each season, nearly two-thirds of individuals 50-64 years with influenza/ILI were employed; 51.7% reported absenteeism in 2018/19 and 53.6% in 2019/20. Of the 20% of individuals 65 years and older who were employed, 47.0% of those with influenza/ILI were absent while ill in 2018/19 (39.8% in 2019/20). In 2018/2019, 29.6% of respondents 50-64 years old with influenza/ILI identified as volunteers (29.3% in 2019/2020). In both seasons, nearly half were unable to do so while ill. Of the 164 (32.7%) individuals 65 years and older who volunteered during the 2018/19 season, 80 (48.8%) did not while ill; 224 (37.3%) respondents volunteered in the 2019/20 season, and half were absent while ill. Of those 50-64 years with influenza/ILI, 97 (42.2%) and 57 (22.2%) were caregivers in 2018/19 and 2019/20, respectively. In 2018/19 and 2019/20, 40 (41.2%) and 28 (49.1%) caregivers were unable to provide care when ill, respectively. Of those with influenza/ILI in the 65 years and older age group, 123 (24.6%) and 162 (27.0%) were caregivers in 2018/19 and 2019/20, respectively. In 2018/19, 18 (14.6%) caregivers with influenza/ILI did not provide care while ill (42 [25.9%] in 2019/20).

**Discussion:**

In Canadian older adults, influenza and ILI had notable impacts on ability to volunteer and provide care across two recent seasons. Optimization of influenza prevention in this population may yield important societal benefits.

## Background

Influenza is a major cause of illness [1], with those 65 years and over at increased risk for complications including cerebrovascular events and pneumonia [2]. Previous research has also demonstrated the impact of influenza on mortality and hospitalization in this age group [3, 4]. Studies about the consequences of influenza are often focused on healthcare resource utilization [5], and there is a gap in understanding how influenza affects the ability of older adults to conduct the routines of daily life. Such knowledge is important for both practitioners and patients as they weigh the risks and benefits of influenza immunization.

While those 50-64 years have not demonstrated the same influenza-related risks as older adults, they comprise a subgroup that typically has a higher rate of chronic conditions and other risk factors than younger populations [6, 7]. Additionally, 50-64 year olds are often in the workforce, and also have responsibilities including volunteering and providing care to others [8, 9]. Therefore, the impact of influenza on daily life duties in this age group affects productivity and absenteeism rates, with consequences extending beyond the ill individual to their families, their employers and the larger society. We conducted surveys during two recent influenza seasons to explore the impact of influenza on the ability to work, volunteer and care-give in the 50 years and over Canadian population.

## Methods

In March 2017, our study team developed a survey of Canadian adults 65 years and older, called *EXamining the Knowledge,* *Attitudes and Experiences of* *Canadian Seniors* *Towards Influenza (EXACT).* This survey, available in English and French, became the first in a series of annual surveys to understand experiences with and impact of influenza [10]. Following the initial dissemination after the 2016/17 influenza season, we decided to extend the eligibility criteria to individuals 50-64 years for future survey iterations. In August 2019 and May 2020, we administered the survey to Canadians adults aged 50-64 years and 65 years and older, to assess individuals’ experiences during the 2018/19 and 2019/20 influenza seasons, respectively*.*

### Survey development

We developed this survey to obtain information on respondent demographics, influenza vaccination history, and experiences with influenza (as diagnosed by a healthcare provider) and undiagnosed influenza-like illness (defined as comprising sore throat, fever, runny nose and cough). Respondents who reported being diagnosed with influenza or having ILI were also asked about the impact of their condition on ability to work (for those working full-time or part-time at the time of their illness), volunteer and provide care to those for whom they are responsible. There were three potential impact levels: no impact, able to conduct responsibilities but at a lower level than usual, absent/not able to conduct responsibilities). The survey included multiple choice and Likert scale of agreement questions, and employed adaptive questioning to reduce respondent burden.

We tested the survey for face validity and content validity in a sample of 10 Canadian adults 65 years and over. Additionally, once the survey was programmed, a member of the study team tested it for technical functionality prior to survey dissemination.

### Recruitment

Canadian market research firm Leger Marketing disseminated the survey to individuals aged 50-64 years and 65 years and older through their online polling panel of 400,000 Canadians. Details regarding the composition of this panel, recruitment of the panel, and strategies to ensure the integrity of the survey data collected have been previously reported [11]. Leger offers financial incentive in the form of points to panel members based on length of completed surveys; these points can then be redeemed for gift cards, Air-miles or other rewards.

The survey was disseminated by Leger to two samples of panel members living in Canada: those 50-64 years, and 65 years and older. Leger emailed a study introduction (describing the study purpose, anticipated completion time of 10-15 minutes, details about the study team, and data storage) and survey link to each of the two samples. The introduction also specified that completion and submission of the survey implied consent. At this time, the survey responses were captured in a Leger database, and no longer accessible to the respondent. Although the data were not collected anonymously by Leger, only de-identified data was provided to the research team. Leger sampled proportionately to province population to achieve a sample size of approximately 1000 individuals 50-64 years of age and 3500 individuals 65 years and older. Sample size details have been previously reported [12].

### Statistical analysis

We analyzed data overall, and by respondent demographics and influenza vaccination status. Analyses were done using STATA 10.0 (2007, StataCorp, LP, College Station, TX).

## Results

Completed surveys were collected between August 10th and 28th, 2019 for the 2018/19 season, and between May 8th and 29th, 2020 for the 2019/20 season.

### Baseline characteristics

50-64 year olds: We surveyed 1006 adults (mean age = 57.4) after the 2018/19 season and 1001 adults (mean age = 57.1) following the 2019/20 season. The response rate was 30% for the 2018/19 season and 31% for the 2019/20 season. We observed similar demographics between seasons, with nearly half of respondents reporting at least one chronic condition or illness which could potentially be exacerbated by influenza. In both seasons, respondents represented all 10 Canadian provinces (territories were not included, Table 1). Vaccination rates against influenza were also similar in both years (44.4% in 2018/19 and 42.1% in 2019/20).

**Table 1:** Baseline characteristics of survey respondents, by age group and influenza season

	50-64 years		65+ years	
	2018/2019 (*n* = 1006)	2019/2020 (*n* = 1001)	2018/2019 (*n* = 3548)	2019/2020 (*n* = 3500)
Characteristic	n (%)	n (%)	n (%)	n (%)
Age				
Mean age (yrs), SD	57.4, 4.2	57.1, 4.1	71.4, 5.2	71.4, 5.3
50-54 years	287 (28.5)	325 (32.5)		
55-59 years	346 (34.4)	332 (33.2)		
60-64 years	373 (37.1)	344 (34.4)		
65 – 74 years			2677 (75.5)	2663 (76.1)
≥ 75 years			871 (24.5)	837 (23.9)
Biological Sex				
Male	504 (50.1)	489 (48.9)	1701 (47.9)	1586 (45.3)
Female	502 (49.9)	512 (51.2)	1845 (52.0)	1913 (54.7)
Intersex	0 (0)	0 (0)	2 (0.05)	1 (0.03)
Gender identity				
Male	497 (49.4)	488 (48.8)	1705 (48.1)	1584 (45.3)
Female	505 (50.2)	511 (51.1)	1838 (51.8)	1909 (54.5)
Other^a^	4 (0.4)	2 (0.2)	5 (0.1)	7 (0.2)
Province				
British Columbia	139 (13.8)	135 (13.5)	460 (13.0)	503 (14.4)
Alberta	105 (10.4)	105 (10.5)	301 (8.5)	296 (8.5)
Saskatchewan	22 (2.2)	26 (2.6)	76 (2.1)	104 (3.0)
Manitoba	41 (4.1)	37 (3.7)	128 (3.6)	120 (3.4)
Ontario	385 (38.3)	385 (38.5)	1390 (39.2)	1321 (37.7)
Quebec	242 (24.1)	240 (24.0)	961 (27.1)	883 (25.2)
New Brunswick	21 (2.1)	24 (2.4)	68 (1.9)	85 (2.4)
Nova Scotia	30 (3.0)	28 (2.8)	106 (3.0)	115 (3.3)
Prince Edward Island	10 (1.0)	5 (0.5)	15 (0.4)	14 (0.4)
Newfoundland and Labrador	11 (1.1)	16 (1.6)	43 (1.2)	59 (1.7)
Location of residence				
City (100,000 people or more)	608 (60.4)	608 (60.8)	2067 (58.3)	2009 (57.4)
Town (1000 to 99,999 people)	323 (32.1)	324 (32.4)	1186 (33.4)	1174 (33.5)
Village (less than 1000 people)	75 (7.5)	69 (6.9)	295 (8.3)	317 (9.1)
Chronic condition				
None	522 (51.9)	516 (51.5)	1310 (36.9)	1252 (35.8)
Diabetes	141 (14.0)	144 (14.4)	658 (18.5)	600 (17.1)
Heart disease	42 (4.2)	47 (4.7)	302 (8.5)	345 (9.9)
Asthma or chronic lung disease other than COPD	84 (8.3)	104 (10.4)	272 (7.7)	347 (9.9)
Blood disorders^b^	28 (2.8)	22 (2.2)	95 (2.7)	97 (2.8)
High or low blood pressure	286 (28.4)	301 (30.1)	1529 (43.1)	1575 (45.0)
COPD	23 (2.3)	21 (2.1)	184 (5.2)	198 (5.7)
Cancer	18 (1.8)	14 (1.4)	177 (5.0)	175 (5.0)
Neurological disorders	34 (3.4)	32 (3.2)	83 (2.3)	86 (2.5)
Kidney disease	11 (1.1)	11 (1.1)	82 (2.3)	94 (2.7)
Significant trouble with memory	20 (2.0)	13 (1.3)	48 (1.4)	37 (1.1)
Liver disease	18 (1.8)	10 (1.0)	26 (0.7)	34 (1.0)
Have had a transplant AND/OR have an immunosuppressive condition AND/OR taking an immunosuppressive medication	7 (0.7)	19 (1.9)	33 (0.9)	44 (1.3)
HIV/AIDS	9 (0.9)	8 (0.8)	5 (0.1)	7 (0.2)

This table summarizes respondents’ demographics and medical history at the time of survey completion

^a^i.e. transgender, non-binary, fluid, two-spirited

^b^Excluding high or low blood pressure

65 years and older: We surveyed 3548 adults following the 2018/2019 season for a response rate of 41%, and 3500 adults following the 2019/2020 season for a 42% response rate. Mean age in both seasons was 71.4 years, with representation from all Canadian provinces. Similar characteristics were observed in both seasons (Table 1). More than two-thirds of respondents had at least one chronic condition. Slightly more than two-thirds of respondents were vaccinated against influenza in each season (68.7% in 2018/2019 and 69.3% in 2019/20).

### Rates of influenza/ILI and related healthcare utilization

**Fig. 1:**
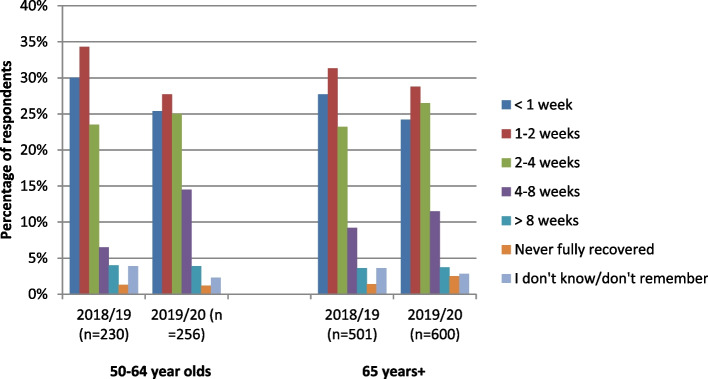
Time until complete recovery from influenza/ILI, by age group and season. Self-reported duration of influenza/ILI varied considerably. For 50-64 year olds, 31.7 and 44.5% had recovery times 2 weeks or longer in 2018/19 and 2019/20, respectively (p < 0.004). In 2018/19, 37.3% of respondents 65 years and older reported taking 2 weeks or longer to recover (44.2% in 2019/20, p < 0.02)

50-64 year olds: Compared to the 2018/19 season, respondents in the 2019/20 season had lower self-reported rates of influenza/ILI (22.9% vs. 25.5%) and higher illness recovery times over two weeks (31.7% vs. 44.5%; *p* < 0.004, Fig. 1).

Those ill with influenza/ILI reported significant healthcare use (Table 2) - during the 2018/19 and 2019/20 seasons, 94 (40.9%) vs. 120 (46.9%) visited a pharmacy, and 57 (24.8%) vs. 53 (20.7%) visited their family physician, respectively. A small percentage required hospitalization or a stay in a long-term care facility while ill (Table 2).

**Table 2:** Healthcare resource utilization while respondent had influenza/ILI, by age group and season

	50-64 years				65+ years			
	2018/2019 (*n* = 230)		2019/2020 (*n* = 257)		2018/2019 (*n* = 501)		2019/2020 (*n* = 600)	
	n (%)	Mean number of visits per person	n (%)	Mean number of visits per person	n (%)	Mean number of visits per person	n (%)	Mean number of visits per person
Pharmacy	94 (40.9)	1.6	120 (46.9)	1.8	188 (37.5)	1.7	262 (43.7)	1.9
Family doctor	57 (24.8)	1.5	53 (20.7)	1.5	151 (30.1)	1.6	160 (26.7)	1.5
Walk-in clinic	37 (16.1)	1.5	29 (11.3)	1.2	57 (11.4)	1.4	62 (10.3)	1.2
Emergency room	14 (6.1)	1.1	12 (4.7)	1.6	18 (3.6)	1.2	32 (5.3)	1.4
Hospitalization	6 (2.6)	LOS: 3.7 days	3 (1.2)	LOS^b^: 5.3 days	7 (1.4)	4.3	8 (1.3)	8
LTC^a^			5 (2.0)	LOS: Ongoing^c^			1 (0.2)	LOS: 10 days
None	99 (43.0)		105 (41.0)		238 (47.5)		277 (46.2)	

Healthcare resource utilization, particularly pharmacy visits and appointments with family physicians, was considerable for both age groups, across 2018/19 and 2019/20

^a^This was not asked in 2018/19

^b^LOS: Length of stay

^c^Ongoing at time of survey

65 years and older: Compared to the 2018/19 season, 2019/20 had higher self-reported rates of influenza/ILI (501 [14.1%] vs. 600 [17.1%], *p* < 0.001) and of recovery times longer than two weeks (187 [37.3%] vs. 265 [44.2%], *p* < 0.02, Fig. 1).

Healthcare resource utilization was common: 188 (37.5%) and 262 (43.7%) visited a pharmacy (*p* < 0.04), while 151 (30.1%) and 160 (26.7%; *p* = 0.20) saw their family physician in 2018/19 and 2019/20, respectively (Table 2).

No statistically significant differences in illness recovery times or healthcare utilization were observed between the two age groups.

### Impact of influenza/ILI on work productivity and absenteeism

50-64 year olds: In each season, nearly two-thirds of individuals with self-reported influenza were employed (143 [62.2%] in 2018/19 and [169] 65.4% in 2019/20). Employed respondents who reported influenza/ILI had comparable absenteeism rates while ill (51.7% reported taking at least one day off work [mean = 4.2 days] in 2018/19 and 53.6% [mean = 4.4 days] in 2019/20), though a significantly higher rate worked at lower capacity while ill in 2018/19 than 2019/20 (55.2% vs. 42.9%, *p* < 0.03) (Table 3).

**Table 3:** Impact of influenza on ability to work, volunteer and provide care, by age group and year

	50-64 years		65+	
	2018/2019	2019/2020	2018/2019	2019/2020
*Individuals with influenza: N*	230	257	501	600
*Working individuals with influenza/ILI*: *n (%)*	143 (62.2)	168 (65.4)	83 (16.6)	113 (18.8)
Absent from work: n (%)	74 (51.7)	90 (53.6)	38 (47.0)	45 (39.8)
Mean number of absent days per person (range)	4.2 (0.5-42)	4.4 (1-30)	4.1 (1-25)	8.2 (1-40)
Present but unable to work at usual capacity: n (%)	79 (55.2)	72 (42.9)	31 (37.3)	46 (40.7)
Mean number of sub-optimal work days per person (range)	3.5 (0.5-14)	4.6 (1-45)	4.0 (1-25)	6.2 (1-30)
*Volunteers with influenza/ILI: n*	68 (29.6)	75(29.3)	164 (32.7)	224 (37.3)
Unable to volunteer: n (%)	34 (50.0)	33 (44.0)	80 (48.8)	111 (49.6)
Mean number of absent hours per person (range)	7.8 (1-24)	7.2 (2-24)	14.4 (1-336)	25.3 (1-1440)
*Caregivers with influenza/ILI: n*	97 (42.2)	57 (22.2)	123 (24.6)	162 (27.0)
Unable to provide care: n (%)	40 (41.2)	28 (49.1)	18 (14.6)	42 (25.9)
Mean number of absent days per person (range)	5.3 (1-14)	6.9 (1-21)	7.8 (1-30)	13.1 (1-72)
Able to provide care but not at usual capacity: n (%)	10 (45.4)	27 (47.4)	52 (42.3)	46 (28.4)
Mean number of sub-optimal caregiving days per person (range)	6.4 (1-21)	6.1 (1-30)	5.8 (1-30)	8.8 (1-80)

Respondents self-reported work, volunteer and caregiving absences while ill with influenza/ILI. Being sick also restricted individuals from being able to optimally fulfill their responsibilities at full capacity

65+: In this age group, in both seasons, the majority of respondents with influenza/ILI were retired (407 [81.2%] in 2018/19 and 472 [78.7%] in 2019/20). Nearly half of those (47.0%) with influenza/ILI who work were absent while ill during the 2018/19 season (39.8% during the 2019/20 season) Table 3.

Of working individuals with influenza/ILI, those who were 50-64 years were more likely to be both absent from work (*p* = 0.009 in 2019/20) or present at work but working at low capacity than older adults (*p* = 0.02 in 2018/19). However, those 65 years and older who were absent from work, were home from work for a longer period of time in 2019/20, and worked at sub-optimal capacity for a slightly longer period of time than the younger age group. These differences were not statistically significant.

### Impact of influenza/ILI on ability to volunteer

50-64 year olds: In 2018/19, 29.6% of respondents with self-reported influenza/ILI identified as volunteers, indicating that they volunteered daily, weekly, monthly or occasionally during that season (29.3% in 2019/20). In both seasons, nearly half were unable to do so while ill with influenza/ILI, with approximately a full day of volunteering lost (mean of 7.8 hours per person in 2018/19 and 7.2 hours in 2019/20; Table 3).

65+: Of the 164 (32.7%) individuals who volunteered at least occasionally during the 2018/19 season, 80 (48.8%) did not while ill; 224 (37.3%) respondents volunteered in the 2019/20 season, and 111 (49.6%) were absent while ill (Table 3). In 2019/20, respondents reported a higher mean number of absent volunteer hours, compared to 2018/19 (25.3 hours vs. 14.4 hours).

No significant differences were observed when comparing impact of influenza/ILI on volunteering, between age groups, although those 65 years and older were absent for a longer mean number of hours than those 50-64 years in both seasons.

### Impact of influenza/ILI on ability to give care

50-64 year olds: Of those with influenza/ILI, 97 (42.2%) and 57 (22.2%) were caregivers in 2018/19 and 2019/20, respectively. Those who provided care most commonly did so for spouse/partner, child, or parent. In 2018/19, 40 (41.2%) were unable to provide care when ill (mean = 5.3 days) while in 2019/20, 28 (49.1%) caregivers who had influenza/ILI could not provide care during illness (mean = 6.9 days) (Table 3).

65+: Of those with influenza/ILI, 123 (24.6%) and 162 (27.0%) were caregivers in 2018/19 and 2019/20, respectively, mainly for spouse/partner, grandchildren and parents. Compared to 2018/19, a higher rate of caregivers did not provide care while ill with influenza/ILI in 2019/20 (18 [14.6%] vs. 42 [25.9%]; *p* < 0.02; mean = 7.8 vs.13.1 days) while fewer provided care at lower capacity than usual (52 [42.3%} vs. 46 [28.4%]; mean = 5.8 vs. 8.8 days; *p* < 0.01) (Table 3).

Of individuals with influenza/ILI who also act as caregivers, those who were 50-64 years were more likely to be both unable to provide care (*p* < 0.001) and able to provide care but at less-than-usual capacity than older adults in 2019/20 (*p* = 0.009).

## Discussion

To better understand the impact of influenza and influenza-like illness on life responsibilities and activities for Canadian adults 50 years and older, we conducted a survey after the 2018/2019 and 2019/2020 influenza seasons. The consequences of influenza/ILI on activities such as working, volunteering and caring for others had not previously been fully explored in this population. We found that approximately four out of 10 individuals 50 years and older with influenza/ILI reported recovery times of longer than two weeks. Additionally, about half reported coming to work but functioning at a lower capacity than usual for approximately four days, and may have transmitted their illness to their colleagues. Nearly half of the respondents who reported volunteering were unable to do so while ill, and were absent for an equivalent of nearly one day. Approximately one-quarter reported caregiving, and approximately one in five were unable to provide care for 1 to 2 weeks while ill with influenza/ILI. Also, 45% of respondents provided care at lower capacity than usual for six additional days. Of those 65 years and older with influenza/ILI, 40% had recovery times of longer than two weeks. Of the one-third of individuals who volunteered during the 2018/19 season, nearly half did not while they were ill, for an average of two to three days. Of those with influenza/ILI, one-quarter were caregivers, and a minority did not provide care while ill with influenza/ILI while a larger percentage provided care but at lower capacity than usual. These results indicate that the impact of influenza extends beyond healthcare resource utilization, and results in productivity losses, and significant societal consequences.

In previous Canadian and U.S. studies that have focused on lost productivity associated with influenza, it has been estimated that two days of work were lost per household [13–15]. In England, community influenza adult cases from 2006 and 2011 resulted in 3.6 days off work lost related to influenza A and 2.4 days for influenza B [16]. Our study indicated more extensive productivity loss which may be attributable to our focus on an older population, and one more likely to have chronic conditions that may lead to more severe illness and longer lasting influenza consequences.

Many studies of productivity losses for caregivers associated with influenza illness have focused on the impact of pediatric influenza on work absence in those who provide care. Studies conducted in the US and England quantifying the impact of influenza on families found that for each child reporting influenza/ILI, mean work absence for caregivers typically ranged from one to four days, and up to 73 hours per illness [17–19]. To our knowledge, this study is the first to examine the impact of influenza on the ability of adults 50 years and older to volunteer and provide care. Although the impact of infection on volunteering and unpaid caregiving is rarely included in the evaluation of potential vaccination impact, the economic value of each is quite significant [20, 21]. A considerable proportion of older adults are involved in providing care to family members, such as partners or grandchildren. A previous study has linked high rates of caregiving with higher rates of respiratory illness including influenza; as expected, the act of regularly providing care to grandchildren is associated with transmission of influenza to grandparents [22]. We have identified that influenza/ILI is associated with a reduction in ability to provide care for up to two full weeks. Unlike perhaps those who work or volunteer, caregivers may not have the option of abstaining from the activity when ill; this appears to be borne out in our findings, where a significant proportion of caregivers still did so while recovering, but were functioning at less capacity than usual. This was particularly true of those 65 years and older, who were more likely to still provide care while ill than younger survey respondents, which may be attributed to them being the sole caregiver and unable to take a break to recover. Caring for dependents while ill has many consequences, both in terms of potentially prolonging recovery, and also placing others at risk for influenza.

Approximately half of our respondents who were employed or who were regular volunteers continued to work or volunteer even while ill with influenza/ILI. This has important implications for both presenteeism and productivity, and even more importantly (as we now have even greater awareness of with COVID-19), for infection control and spread of viral illnesses. We found a statistically significant decrease in these behaviours and an increase in absenteeism in the 2019/20 season versus the 2018/19 season, which may be indicative of a more severe influenza season, given that we also noted increased healthcare utilization in the 2019/20 season. Additionally, it may also be reflective of the awareness of public health messaging to stay home while ill that was growing in the later part of the 2019/20 season due to the onset of the COVID-19 pandemic.

In comparing older vs. younger respondents, we found that the impact on life responsibilities and activities was fairly similar. However, having influenza/ILI was more likely to result in absenteeism from work and caregiving for the 50-64 year adults than the older age group. This highlights the importance of targeted messaging about influenza prevention, including through vaccination, to adults 50 years and older who may not see themselves at risk of the daily impact from influenza.

This study has several strengths including the large number of respondents, inclusion of all 10 Canadian provinces, and samples similar to the country’s senior population in terms of chronic illness prevalence [23]. Additionally, we benefited from collecting data after two influenza seasons that had differing severities. The 2018/19 influenza season was unusually prolonged in Canada, and was characterized by two waves of Influenza A (the first, and larger, wave being H1N1 with a second smaller H3N2 wave [24]. The 2019/20 influenza season was also A/H1N1 dominant, and included the beginning of the COVID-19 pandemic which was associated with a dramatic drop off in influenza circulation in late March 2020 [25].

Our study has some limitations, several previously described [10]. Our online sampling frame may have excluded Canadians who are very ill, cognitively impaired, or uncomfortable with technology. Given that we are asking respondents to self-report about illness that happened during the influenza seasons, recall bias cannot be excluded. We attempted to limit the effect of this by being explicit about the timeframe we were inquiring about and giving respondents the ability to respond “I don’t know/I don’t remember”. It is also possible that rates of self-reported ILI during the 2019/20 season were overestimated given that respondents who assumed their undiagnosed illness was influenza may have actually had COVID. Our survey response rate across the two adult populations in both years was 30-41%, and as is true of all surveys, those who responded may have different opinions or experiences from those who did not. To understand the extent of this potential bias, we have compared our results to those reported in the literature (in 2020, rates were 39.8% for 50-64 year olds and 64.7% for those 65 years and older), and have noted similarities although our immunization rates were slightly higher [26]. Finally, we did not collect antiviral receipt data, although having this information may have been useful in understanding recovery rates.

## Conclusions

Our findings provide important insight on the notable impacts of influenza on both healthcare resource utilization and ability to conduct daily life including reduced ability to work, volunteer and provide care to dependents, for those 50 years and older. Such data can inform discussions of the risks and benefits of influenza vaccination and contribute to more comprehensive economic and social evaluations of immunization programs.

## Data Availability

The datasets generated and analysed during the current study are not publicly available given that study participants did not consent for their individual data to be shared (aggregate data sharing only), and the possibility that a subset of participants may be identified from their responses which would compromise anonymity.
